# Potential antibacterial, antibiofilm, and photocatalytic performance of gamma-irradiated novel nanocomposite for enhanced disinfection applications with an investigated reaction mechanism

**DOI:** 10.1186/s12866-023-03016-3

**Published:** 2023-09-26

**Authors:** Gharieb S. El-Sayyad, M. Abd Elkodous, Hanan S. El-Bastawisy, Waleed M. A. El Rouby

**Affiliations:** 1 Drug Radiation Research Department, National Centre for Radiation Research and Technology (NCRRT), Egyptian Atomic Energy Authority (EAEA), Cairo, Egypt; 2 Center for Nanotechnology (CNT), School of Engineering and Applied Science, Nile University, Sheikh Zayed, Giza, 16453 Egypt; 3 Material Science and Nanotechnology Department, Faculty of Postgraduate Studies for Advanced Sciences (PSAS), Beni-Suef University, Beni-Suef, 62511 Egypt

**Keywords:** CdS NPs, Antibacterial activity, Photocatalysis, Bacterial membrane leakage, Disinfection, Methylene blue

## Abstract

**Background:**

Water scarcity is now a global challenge due to the population growth and the limited amount of available potable water. In addition, modern industrialization, and microbial pathogenesis is resulting in water pollution on a large scale.

**Methods:**

In the present study, reusable Co_0.5_Ni_0.5_Fe_2_O_4_/SiO_2_/TiO_2_ composite matrix was incorporated with CdS NPs to develop an efficient photocatalyst, and antimicrobial agents for wastewater treatment, and disinfection purpose. The antibacterial performance of the gamma-irradiated samples was evaluated against various types of Gram-positive bacteria using ZOI, MIC, antibiofilm, and effect of UV-exposure. Antibacterial reaction mechanism was assessed by bacterial membrane leakage assay, and SEM imaging. In addition, their photocatalytic efficiency was tested against MB cationic dye as a typical water organic pollutant.

**Results:**

Our results showed that, the formed CdS NPs were uniformly distributed onto the surface of the nanocomposite matrix. While, the resulted CdS-based nanocomposite possessed an average particle size of nearly 90.6 nm. The antibacterial performance of the prepared nanocomposite was significantly increased after activation with gamma and UV irradiations. The improved antibacterial performance was mainly due to the synergistic effect of both TiO_2_ and CdS NPs; whereas, the highest photocatalytic efficiency of MB removal was exhibited in alkaline media due to the electrostatic attraction between the cationic MB and the negatively-charged samples. In addition, the constructed heterojunction enabled better charge separation and increased the lifetime of the photogenerated charge carriers.

**Conclusion:**

Our results can pave the way towards the development of efficient photocatalysts for wastewater treatment and promising antibacterial agents for disinfection applications.

**Supplementary Information:**

The online version contains supplementary material available at 10.1186/s12866-023-03016-3.

## Background

The world is now facing a global water shortage problem due to either the tiny portion of accessible potable water on earth (about 0.9%) or due to the large amount of wasted or polluted water [1–3].

In addition, polluted water is a host for many pathogenic microorganisms causing serious diseases like hepatitis A, diarrhea, and typhoid fever [4, 5]. The tremendous expense of their prevention and treatment, as well as the morbidity and death they cause, make waterborne pathogens and associated disorders a major public health problem across the world [6, 7]. These illnesses have a direct connection to pollution and environmental degradation. Waterborne outbreaks are continuously being reported internationally despite ongoing efforts to preserve water safety [8, 9]. In order to make decisions on the infrastructure of water distribution systems, the best water treatment, and the prevention of waterborne epidemics, an accurate evaluation of pathogens in water and monitoring of water quality are essential [10, 11].

Depending on an array of geographical, socioeconomic, seasonal, and microbiological circumstances, the relative significance of the quality of drinking water to the preservation of public health may change [9, 12]. It is impossible to definitively say which feature of water availability is more crucial at any given moment or in any given place. But it's becoming more and more obvious that every aspect of drinking-water availability and quality has the potential to be significant and has to be taken into account [11]. In this context, it is important to emphasize that one of the few generalizations regarding drinking-water quality is that endemic or pandemic waterborne illness will not emerge if faecal-derived pathogens are absent [9, 13].

Remains of antibiotics in the environment of water may cause environmental bacteria to become resistant, which might then spread to pathogens and make the treatment of illnesses more challenging, posing a severe danger to public health. Antibiotic resistance may be lowered if environmental antibiotic residues could be removed or reduced. To achieve this goal, antibiotics-containing water was treated using Fe-doped ZnO nanoparticles in sunlight-assisted photocatalysis to determine the system's capacity for degradation [14].

Consequently, developing new technologies for wastewater treatment is significant nowadays [15–17]. Currently, there are many methods for water treatment which can be classified into three main categories: physical, chemical, and biological processes [18, 19]. Among them, heterogeneous photocatalysis through nano-sized semiconductor photocatalyst is a cost-effective, fast, and efficient method for water treatment [18, 20–22]. This technique relies on the redox reactions occurred on the surface of the semiconductor photocatalysts after absorbing light of energy equal to or higher than their bandgap [23, 24]. Many metals oxide-based semiconductors (ex: ZnO, WO_3_, SnO_2_, and CeO_2_) were employed as photocatalysts for water treatment and microbial disinfection [14, 25, 26]. With respect to all semiconductor photocatalysts, anatase TiO_2_ NPs photocatalyst is still attracting the attention of many researchers worldwide because of its abundance, higher chemical and thermal stabilities, non-toxicity nature, and cost-effectiveness [27, 28]. Interestingly, TiO_2_ NPs have both good photocatalytic and antimicrobial properties making it a perfect candidate for water treatment applications [29–31]. TiO_2_ and doped with noble metals are hence suitable choices for these applications [32]. TiO_2_ has unique physical and chemical properties that are dependent on the crystal phase, particle size, and shape. For instance, the band gaps of different crystalline phases of TiO_2_, such as rutile TiO_2_'s band gap of 3.0 eV and anatase TiO_2_'s band gap of 3.2 eV, affect the material's capacity as a photocatalyst [33]. However, the photocatalytic efficiency of TiO_2_ NPs is hampered by their large bandgap and the rapid recombination of their photogenerated charge carriers [34, 35]. Also, it demonstrates electrical, optical and morphological properties which make TiO_2_ preferable for environmental applications [36, 37]. In addition, nanoparticles (NPs) are less functional than nanocomposites which gather the merits of many NPs. That’s the reason why many techniques were developed to tailor the limitations of TiO_2_ NPs including doping with Noble or non-Noble metal ions and conjugation with other semiconductor, carbon materials, or plasmonic metals to construct heterojunctions [38, 39]. The constructed heterojunction plays a significant role in charge separation, elongating the lifetime of photogenerated charge carriers, and increasing the visible light absorption. One of the promising heterojunctions is the semiconductor–semiconductor heterojunction established between TiO_2_ and CdS NPs. CdS NPs are not only promising in technological applications, but also in biomedical uses due to their antimicrobial potential [40, 41]. Post-treatment procedures have also been investigated as a way to improve the characteristics of metal oxide NPs. For instance, it has been demonstrated that being exposed to extremely powerful ionizing radiations, such as X-rays, and gamma radiation, results in major modifications in the microstructural and morphological characteristics of metal oxides and generates a wide range of defects, which can change their optical, electrical, catalytic, antimicrobial, and sensing properties [42, 43].

In the current study, layer-by-layer method was used to prepared recyclable Co_0.5_Ni_0.5_Fe_2_O_4_/SiO_2_/TiO_2_ composite matrix, which was decorated by CdS NPs using impregnation method, to construct a semiconductor–semiconductor heterojunction between (TiO_2_ and CdS NPs). Then, the prepared samples were gamma-irradiated and their antimicrobial performance against multidrug-resistant pathogenic bacteria was evaluated using many assays (ZOI, MIC, antibiofilm, and effect of UV-exposure). Antibacterial reaction mechanism was assessed by bacterial membrane leakage assay, and SEM imaging. In addition, their photocatalytic efficiency against cationic methylene blue (MB) dye was evaluated under different pH values.

## Materials and methods

### Materials

Nickel chloride, cobalt chloride, ferric chloride, tetraethyl orthosilicate 98% (TEOS), absolute ethanol, hydroxypropyl cellulose (M.W. = 80,000), ammonium hydroxide 28%, titanium (IV) isopropoxide 97%, sodium hydroxide, hydrochloric acid, thiobenzoic acid, ether, 1-Thioglycerol, chloroform, sodium bicarbonate, acetonitrile, cadmium acetate, 2-propanol, and 2, 2-bipyridine were used as received from Fluka, Sigma Aldrich, Wako, and Merck (Germany—Japan). All chemicals were of extra pure grade. Other chemicals and media used in the microbiological methods were of pure grade and used as received without any further purification. All the solutions were prepared using distilled water (DW).

### Preparation of composite matrix (Co_0.9_Ni_0.1_Fe_2_O_4_/SiO_2_/TiO_2_)

Composite matrix used in this study was prepared using a Layer-by-layer method and the detailed steps are mentioned in our early-published articles [1, 5].

### Preparation of cadmium sulfide nanoparticles (CdS NPs)

CdS NPs were prepared using a method reported by Zhang *el al.,* with modifications [44]. Briefly, (10 mM) of 1-thioglycerol was dissolved in (25 mL) D.I.W., then solution pH was adjusted to 11 using (1 M) NaOH. After that, the formed solution was inserted into a three-necked flask containing septum and valve. Then, a certain volume of the previously-prepared [(2, 2′-bpy) Cd (SCPh)_2_] precursor was added to the above solution. Subsequently, N_2_ bubbling was used for the de-aeration of the resulted solution, which was refluxed for 20 min at 100 °C. Pale yellow solution was formed indicating the formation of CdS NPs after the decomposition of the precursor. Finally, the formed particles were collected using centrifugation, washed with D.I.W. several times, and dried at 60°C.

### Preparation of CdS-loaded nanocomposite (Co_0.9_Ni_0.1_Fe_2_O_4_/SiO_2_/TiO_2_/CdS)

The prepared CdS NPs were uniformly-distributed onto the surface of the prepared composite matrix via a simple impregnation route. Firstly, 250 mg of the prepared composite matrix was dispersed in 50 mL of super dehydrated ethanol solution using water bath sonication for 30 min. at room temperature. Secondly, about 30 mg of the prepared CdS NPs were added into the dispersion, which was left overnight under vigorous stirring at room temperature. Finally, the precipitate was collected by centrifugation (5000 rpm), washed several times using D.I.W. and dried at 60°C in air.

### Characterization methods

The size and morphology of CdS-loaded nanocomposite were inspected by high resolution transmission electron microscopy (HRTEM, JEOL 3010, Japan) operated at 200 kV. The surface structure and homogeneity of the synthesized CdS-loaded nanocomposite were characterized by scanning electron microscopy (SEM) ZEISS, EVO-MA10 equipped with Energy-Dispersive X-ray spectra (EDX) for elemental analysis.

### Antibacterial activity of CdS, and CdS-loaded nanocomposite

The antimicrobial potential of as-synthesized CdS, and CdS-loaded nanocomposites against different pathogenic bacteria are examined via employing the agar-well diffusion method. Firstly, the as-synthesized CdS, and CdS-loaded nanocomposites are dissolved into dimethyl sulfoxide (DMSO; 10%) with different concentrations (0.01 and 0.02 mg/ml; 10, and 20 ppm, respectively). Additionally, the CdS-loaded Co_0.9_Ni_0.1_Fe_2_O_4_/SiO_2_/TiO_2_ nanocomposite was irradiated with gamma rays at various doses (25.0, 50.0 and 100.0 kGy) in order to investigate their effect on its antimicrobial activity.

The activity of the as-synthesized compounds was examined upon several isolates of pathogenic bacteria afforded from the culture collections in Drug Microbiology Lab., NCRRT, Cairo, Egypt. The pathogenic bacteria were separated from surfaces and exterior in the medical operating places (such as beds, surfaces, walls, and doors). The tested bacterial isolates were distinguished, and identified in our earlier study [45], also, they were tested for their ability for biofilm formation.

Bacterial strains were *Staphylococcus lentus*, *Staphylococcus sciuri*, *Staphylococcus vitulinus*, *Staphylococcus aureus*, *Aerococcus viridians*, and *Enterococcus columbae*. The bacterial inoculums were fixed at 0.5 McFarland (1–3) × 10^8^ CFU/ml, applying fixed 600 nm UV–Vis. spectrophotometer [46]. The growth restraint of all the investigated bacterial strains was estimated by the zone of inhibition (ZOI) after 24 h incubation. Conventional antibiotic discs (Amoxicillin (AX; 25 μg/ml; 6.0 mm diameter), was chosen to determine the performance of the synthesized compounds [47].

The minimum inhibitory concentrations (MIC) investigation is completed in Luria–Bertani (LB) broth within a serial dilution. Briefly, a positive control (the microorganism and the nutrient), a negative control (the nutrient solely), and the examined CdS, and CdS-loaded composites (beginning with 0.1 mg/ml concentration; 100 ppm) are applied; MIC is defined following 24 h at 37° C. The inoculums of the tested bacteria are at 3–5 × 10^8^ CFU/ml. MIC is defined by operating ELISA plate reader at a specific wavelength (600 nm) [48]. Finally, the results are statistically analyzed by applying ONE WAY ANOVA, the least significant difference (LSD), and Duncan's multiple ranges, which are calculated by special software (SPSS version 15).

### Antibiofilm activity of CdS-loaded nanocomposite against the isolated bacteria

Tissue culture plate method (TCP) is the most widely assay used to detect the biofilm formation. All isolates were screened for their ability to form biofilm as described by the standard reference [49]. Overnight-grown cultures were diluted 1: 100 in brain heart infusion (BHI) broth and incubated for 2 h in shaking conditions to bring it to the exponential-growth phase. The cultures were further diluted in BHI broth to attain a bacterial density of 1.5 × 10^8^ CFU ml^−1^. 100 μl of 100 kGy (ɤ irradiated) CdS-loaded nanocomposite was added to each well of 96 well microtiter plate containing 100 μl of each tested bacterial suspensions, incubate at 37°C for 18–24 h. Then the supernatant was discarded and washed with Phosphate Buffer Saline (PBS), adherent organisms were fixed by incubation for 1h at 60°C. The OD values of the adhered isolates were measured by using ELISA reader after staining with crystal violet (1%) and decolorizing with 200 μl 95% (v/v) ethanol. Biofilm producers were classified as negative (non-adherent), weak (weakly adherent), and high (strongly -adherent) biofilm producers according to the observed OD values, results were compared with the control non-treated one to investigate the efficiency of CdS-loaded nanocomposite as biofilm inhibitor. The biofilm inhibition percentage (%) was determined according to the following equation:

$$\mathbf B\mathbf a\mathbf c\mathbf t\mathbf e\mathbf r\mathbf i\mathbf a\mathbf l\boldsymbol\;\mathbf b\mathbf i\mathbf o\mathbf f\mathbf i\mathbf l\mathbf m\boldsymbol\;\mathbf i\mathbf n\mathbf h\mathbf i\mathbf b\mathbf i\mathbf t\mathbf i\mathbf o\mathbf n\boldsymbol\;\boldsymbol\%=\frac{\mathbf O.\;\mathbf D.\;\mathbf o\mathbf f\boldsymbol\;\mathbf t\mathbf h\mathbf e\boldsymbol\;\mathbf c\mathbf o\mathbf n\mathbf t\mathbf r\mathbf o\mathbf l\boldsymbol\;\mathbf s\mathbf a\mathbf m\mathbf p\mathbf l\mathbf e-\mathbf O.\;\mathbf D.\;\mathbf o\mathbf f\boldsymbol\;\mathbf t\mathbf h\mathbf e\boldsymbol\;\mathbf t\mathbf r\mathbf e\mathbf a\mathbf t\mathbf e\mathbf d\boldsymbol\;\mathbf s\mathbf a\mathbf m\mathbf p\mathbf l\mathbf e}{\mathbf O.\;\mathbf D.\;\mathbf o\mathbf f\boldsymbol\;\mathbf t\mathbf h\mathbf e\boldsymbol\;\mathbf c\mathbf o\mathbf n\mathbf t\mathbf r\mathbf o\mathbf l\boldsymbol\;\mathbf s\mathbf a\mathbf m\mathbf p\mathbf l\mathbf e}\times100$$

### Effect of UV irradiation

The antibacterial activity of the as-synthesized 100 kGy (ɤ-irradiated) CdS-nanocomposite with and without UV illumination was assessed against the tested pathogenic bacteria using the optical density method. The tested bacteria were stimulated in nutrient broth (NB) overnight at 37°C. Firstly, 0.5 ml of the overnight culture were inoculated to 5 ml NB tubes that adjusted after 2 h of incubation to standard 0.5 McFarland concentration that standardly equals 1.5 × 10^8^ CFU of bacteria. 100 µL of 100 kGy ɤ irradiated CdS-nanocomposite were added into the tubes and then incubated at 37˚C for 60 min. Tubes were grouped into four groups; (1) Tubes without 100 kGy ɤ-irradiated CdS-nanocomposite and displayed to UV illumination were used as a positive control, (2) tubes without UV illumination were used as negative control and does not contains CdS-nanocomposite, (3) tubes with 100 kGy ɤ irradiated CdS-nanocomposite and not UV illuminated, and (4) tubes with 100 kGy ɤ irradiated CdS-nanocomposite and UV illumination. After the incubation, the turbidity of the medium was measured at 600 nm for bacteria.

### Growth curve assay

The influence of non-irradiated and irradiated CdS-loaded nanocomposites on the growth of *S. aureus* (the most sensitive microbes) was determined by the growth curve assay according to Huang et al*.,* [50]. The bacterial suspension was adjusted to 0.5 McFarland (1 × 10^8^ CFU/ml) in 5.0 ml of nutrient broth tubes. Equal volumes of non-irradiated and irradiated CdS-loaded nanocomposites were included separately to every of the examined tubes. The absorbance of the bacterial growth following treatment was evaluated each two hours' time intervals up to 24 h (Wavelength of 600 nm). The average of duplicate readings was considered against the hour periods to get the regular growth curve.

### Effect of the synthesized nanocomposites on protein leakage from bacterial cell membranes

Pure 18 h bacterial culture was set at 0.5 McFarland (1 × 10^8^ CFU/ml) and 100 µl was injected into 10 ml of the nutrient broth include well-sonicated and dispersed non-irradiated and irradiated CdS-loaded nanocomposite at various concentrations (0.125, 0.25, 0.5, and 1.0 mg/ml). Nanocomposites-free broth injected with culture had been used as the control. All the treated samples were incubated at 37°C for 5 h. and then centrifuged or 15 min. at 5000 rpm [51]. For the different samples, 100 μl supernatant had combined with 1 ml of Bradford reagent. Optical density had measured at 595 nm next 10 min of dark incubation [51].

### Reaction mechanism using SEM analysis of CdS-nanocomposite-treated microbial cells

The sensitive microbial cells (from the antibiofilm analysis) were washed with PBS three times and eventually fixed with 4.0% glutaraldehyde solution [52]. The preserved microbial cells were regularly cleaned with PBS and repeatedly drained with various ethanol concentrations (30, 50, 70, 90, and 100%) for 15 min at 28 ± 2°C [53]. Next to that, the fixed samples were solidified on an aluminum portion, considering SEM analysis. The morphological characteristics of the control (non-treated microbial cell), and irradiated CdS-nanocomposite-treated microbes were observed by SEM examination.

### Photocatalytic degradation of methylene blue (MB)

The photocatalytic degradation studies of the synthesized CdS-loaded nanocomposite for degrading methylene blue (MB) were performed with batch equilibrium method [54]. A stock solution (100 mg/l) of MB was prepared by dissolving 0.1 g of MB powder in de-ionized water, and experimental solutions of desired MB concentrations were obtained by successive dilutions of the stock solution with de-ionized water. (5 mg) of the prepared CdS-loaded nanocomposite obtained were added to (50 ml) of an aqueous solution of MB with initial concentration (C_o_ = 20 mg/L), under constant stirring at ambient temperature (24.0 ± 2°C) for 30 min in dark, until adsorption–desorption equilibrium is attained between MB and the prepared photo-catalyst (nanocomposite).

The used UV reactor was cylindrical shape having dimensions of 15 cm length, and 5 cm diameter and made up of glass. The photo-reactor filled with 50 ml of contaminated solutions. The light source of a 400 W halogen lamp where infrared and near-infrared parts of the spectrum contain the majority of the emitted energy (up to 85 percent), with 15–20 percent falling into the visible (400 to 700 nm) and less than 1% in the ultraviolet wavelengths (below 400 nm). The lamp was fixed on about 50 cm distance away from the dye surface where the light intensity about 1 sun. At constant time intervals of irradiation, 1.5 ml was withdrawn and centrifuged at 10000 rpm to separate the photo-catalyst. The intensity of MB absorbance beak (λ_max_ = 664 nm) was measured and the degradation rate of MB was calculated by determining the variation in MB concentration verses irradiation time, by a UV–Vis. spectrophotometer (shimadzu 3600 UV–Vis). DI water was used as a reference [54].

## Results and discussion

### Characterization of the synthesized CdS NPs, and CdS-loaded nanocomposite

#### Scanning electron microscopy

**Fig. 1:**
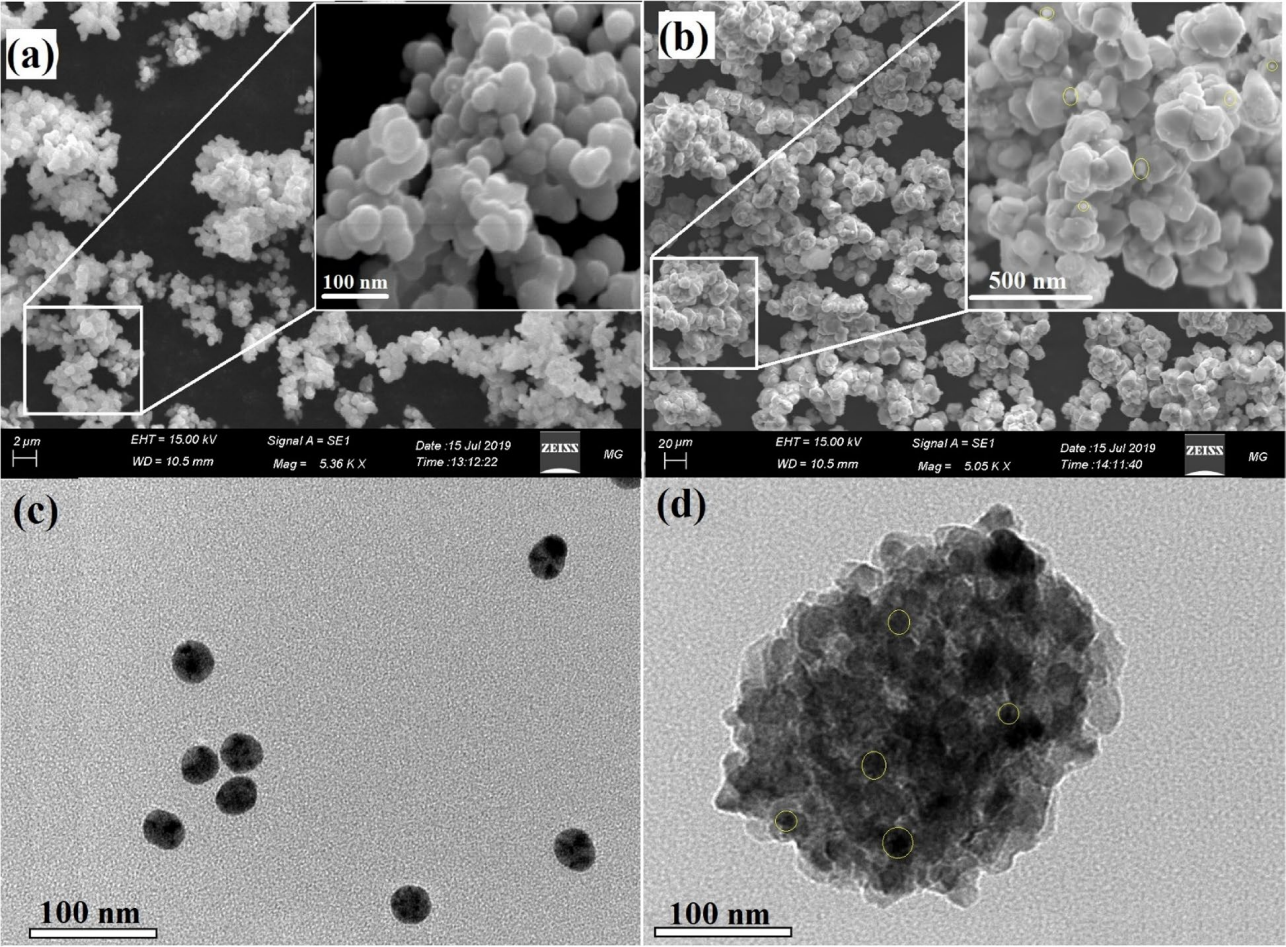
Surface morphology, particle size, and shapes of the synthesized CdS NPs, and CdS-loaded nanocomposite, where **a** SEM of CdS NPs, **b** SEM of CdS-loaded nanocomposite, **c** HRTEM of CdS NPs, and **d** HRTEM of CdS-loaded nanocomposite

SEM was used to examine the formation and surface morphology of the nanocomposite [20]. SEM images of the fabricated CdS NPs, and CdS-loaded nanocomposite are presented in Fig. 1. The synthesized CdS NPs are appeared as a bright aggregate with a spherical surface appearance as noted in Fig. 1a, where the synthesized CdS-loaded nanocomposite are appeared as a fine NPs (yellow circle) located in the surface of the synthesized nanocomposite. It must be mentioned that, the synthesized nanocomposite composite of core ferrite, and the following two layers of SiO_2_ and TiO_2_ NPs are shells around this core, producing a core–shell system as validated in our published papers [1, 5, 20, 55].

#### High-resolution transmission electron microscopy

HRTEM images of the synthesized CdS NPa, and CdS-loaded nanocomposite are presented in Fig. 1. The synthesized CdS NPs are displayed as a separated spherical particle with diameters varying from 45.58 nm to 60.25 nm, and an average diameter of 50.12 nm as shown in Fig. 1c. On the other hand, the integrated CdS-loaded nanocomposite has a semi-spherical construction loaded with a fine oval condensed particle (CdS NPs) with diameters varying from 45.85 nm to 170.58 nm, and an average diameter of 98.58 nm as noted in Fig. 1d, with a detailed average size distribution graph are noted in Figure S1. It is worth noting that the CdS NPs (yellow circles) are assigned to the condensed black particle on the surface of the synthesized nanocomposite. Also, the hazy faint layers are attributed to the basic synthesized nanocomposite.

#### Energy dispersive X-ray spectroscopic analysis

**Fig. 2:**
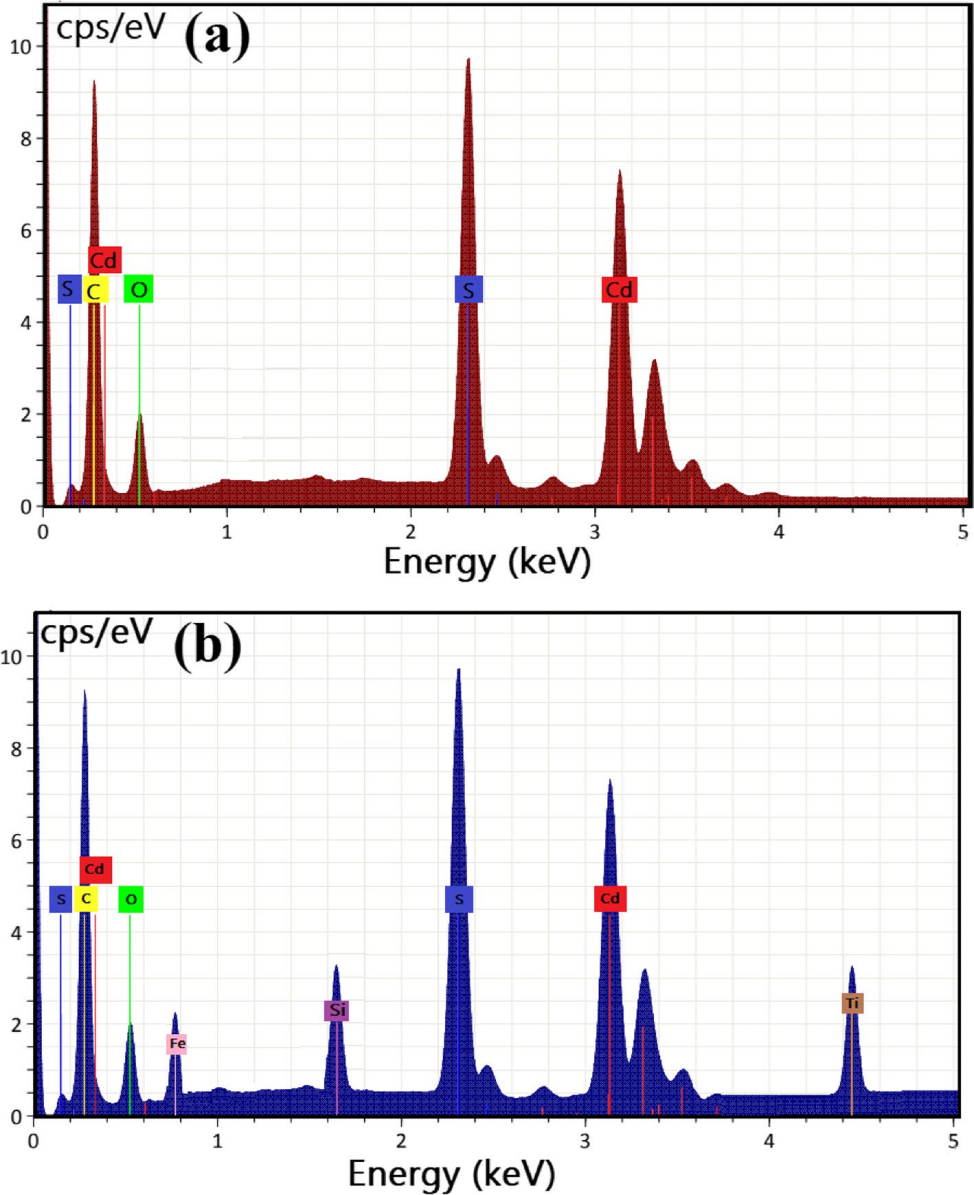
Elemental analysis of the synthesized CdS NPs, and CdS-loaded nanocomposite, where (**a**) EDX of CdS NPs, and (**b**) EDX of CdS-loaded nanocomposite

EDX examination was performed for elemental analysis and purity estimation [20]. Figure 2 showed the EDX spectra of the synthesized CdS NPa, and CdS-loaded nanocomposite. It can be seen that the presence of all elements entire the CdS NPs (such as Cd, S, O, and C) was presented with stoichiometric ratios as sheen in Fig. 2a. At the same time, the appearance of the carbon (C) peak is attributable to the carbon holder for sample preparation in EDX analysis [7, 56]. On the other hand, EDX spectra of the synthesized CdS-loaded nanocomposite (Fig. 2b), indicating the presence of all elements entire the CdS NPs (such as Cd, and S), also Fe for the core ferrite, and both Si, and Ti for the outer shells in the synthesized Co_0.5_Ni_0.5_Fe_2_O_4_/SiO_2_/TiO_2_ nanocomposite, and the absence of both Co, and Ni in the tested sample is due to the disappear of Co, and Ni in the core structure.

### Antibacterial potential

**Fig. 3:**
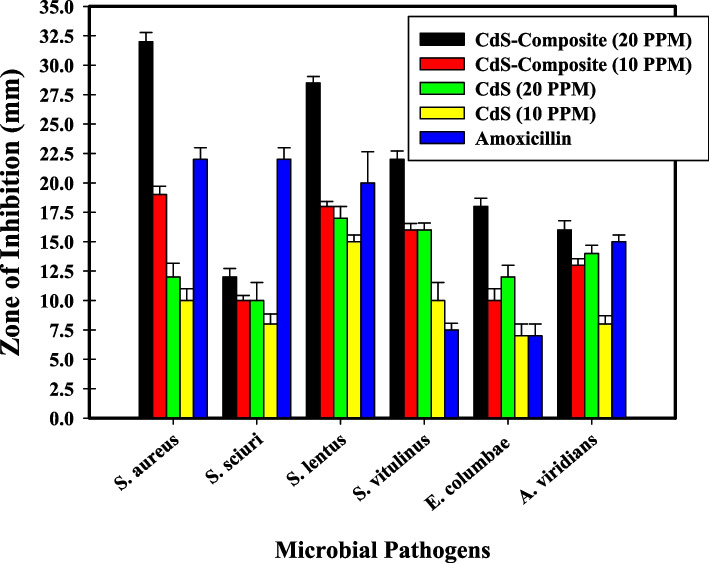
Antibacterial activity of bare CdS NPs and CdS-Co_x_Ni_1-x_Fe_2_O_4_/SiO_2_/TiO_2_ NPs, at different concentrations (20, and 10 μg/mL (PPM)) against some pathogenic bacteria as ZOI (mm)

Over time, It was established that antimicrobial agents were employed to inhibit multiple microbial diseases causing clinical poisoning [57]. However, the idea of investigating new agents based on specific nanomaterials for microbial inhibition is receiving attention [58]. In the present study, the as-prepared nanomaterials were tested for their antimicrobial potential using the well agar diffusion method. Our results indicated that, the developed CdS-nanocomposite (20 ppm) exhibits a deactivation effect representing a wide spectrum against the tested bacteria compared with bare CdS (20 ppm). Additionally, CdS-nanocomposite (20 ppm) showed the most powerful impact against all examined microbes, as shown in Table 1, and Fig. 3.

**Table 1:** Antibacterial activity of bare CdS NPs and CdS-Co_x_Ni_1-x_Fe_2_O_4_/SiO_2_/TiO_2_ NPs, against some pathogenic bacteria as ZOI (mm) and MIC (μg/ml)

**Pathogenic bacteria**	**ZOI of CdS-Co**_**x**_**Ni**_**1-x**_**Fe**_**2**_**O**_**4**_**/SiO**_**2**_**/TiO**_**2**_** NPs (10 ppm) (mm)**	**ZOI of CdS-Co**_**x**_**Ni**_**1-x**_**Fe**_**2**_**O**_**4**_**/SiO**_**2**_**/TiO**_**2**_** NPs (20 ppm) (mm)**	**MIC of CdS-Co**_**x**_**Ni**_**1-x**_**Fe**_**2**_**O**_**4**_**/SiO**_**2**_**/TiO**_**2**_**NPs (µg/ml)**	**ZOI of CdS NPs (10 ppm) (mm)**	**ZOI of CdS NPs (20 ppm) (mm)**	**MIC of CdS NPs (µg/ml)**	^g^**AX**
*Staphylococcus sciuri*	10.0^a^ ± 0.5000	12.0^a^ ± 0.5000	12.5	8.0^ab^ ± 0.1527	10.0^a^ ± 0.3214	25.0	22.0
*Staphylococcus vitulinus*	16.0^c^ ± 0.2886	22.0^d^ ± 0.2886	3.125	10.0^cd^ ± 0.2886	16.0^e^ ± 0.4618	12.5	7.5
*Enterococcus columbae*	10.0^a^ ± 0.2645	18.0^c^ ± 0.2645	6.25	7.0^a^ ± 0.2886	12.0^b^ ± 0.4618	25.0	7.0
*Aerococcus viridians*	13.0^b^ ± 0.5196	16.0^b^ ± 0.5196	6.25	8.0^b^ ± 0.2886	14.0^d^ ± 0.5196	12.5	15.0
*Staphylococcus lentus*	18.0^d^ ± 0.2886	28.0^e^ ± 0.2886	0.390	15.0^e^ ± 0.2886	17.0^f^ ± 0.4163	6.25	20.0
*Staphylococcus aureus*	19.0^e^ ± 0.2645	32.0^f^ ± 0.2645	0.195	10.0^c^ ± 0.5507	12.0^bc^ ± 0.4163	12.5	22.0

Values are means ± SD (*n* = 3)

Data within the groups are analyzed using a one-way analysis of variance (ANOVA) followed by^a, b, c, d, e, f^ Duncan’s multiple range test (DMRT)

^g^*AX* Amoxicillin (antibacterial standard)

The results showed that our samples were more active than the commonly used standard antibiotics and that the some tested microbes were resistant to the used antibiotics. The antimicrobial properties of the as-synthesized nanocomposites were assessed with a conventional antibiotic like Amoxycillin (AX; 25.0 g/ml), and the results indicated that these findings. Inorganic NPs often have a high surface-to-volume ratio, a variety of properties, and a small size. In order to integrate and interact with certain of the infectious microorganisms such as yeasts and bacteria, they can exhibit unique and significant behavior [59].

The exceptional qualities of inorganic NPs make them useful in a variety of medicinal applications and lower the efficacy of conventional antibiotics by making some bacteria more resistant, which decreases the likelihood that they may be used as treatments [60].

The results of MIC were ranged from 0.195 to 12.5 μg/ml of the integrated samples against all the tested microbes. The promising MIC of CdS-Co_x_Ni_1-x_Fe_2_O_4_/SiO_2_/TiO_2_ NPs was 0.195 μg/ml (*S. aureus),* and 0.390 μg/ml (*S. lentus)*.

Importantly, the encouraging properties of the synthesized CdS-Co_x_Ni_1-x_Fe_2_O_4_/SiO_2_/TiO_2_ NPs play a vital role in their antimicrobial characteristics; elemental structure, the purity, and size for the synthesized nanocomposites should be analyzed to explain their antimicrobial pursuits [61]. There is significance within the properties of the prepared CdS-Co_x_Ni_1-x_Fe_2_O_4_/SiO_2_/TiO_2_ NPs and the outstanding antimicrobial performance. The CdS-Co_x_Ni_1-x_Fe_2_O_4_/SiO_2_/TiO_2_ NPs composition and their hopeful particle size presented an important part in improving the antimicrobial efficacy of the developed CdS-Co_0.5_Ni_0.5_Fe_2_O_4_/SiO_2_/TiO_2_ NPs, too at very low concentrations (20.0 µg/ml), facing all established bacteria.

They maintained proper physical and chemical qualities beyond the usual organic and synthetic antimicrobial agents such as a more special link for interactions, deducting for greater synergy with more further pathogenic bacteria and yeast, accordingly-heightening their antimicrobial potential [62].

The antimicrobial mechanism of CdS-Co_x_Ni_1-x_Fe_2_O_4_/SiO_2_/TiO_2_ NPs is however not identified. There were amazing advanced mechanisms such as Reactive Oxygen Species (ROS) distribution (superoxide anion; O_2_^−^) [63], the combination of CdS-Co_x_Ni_1-x_Fe_2_O_4_/SiO_2_/TiO_2_ NPs within the pathogenic microbes and an alkaline tendency were included to demonstrate the antimicrobial activity mechanism [64]. It is suggested, CdS-Co_x_Ni_1-x_Fe_2_O_4_/SiO_2_/TiO_2_ NPs could alter the microbial morphology and their film composition, change the microbial membrane permeability and produce the residence of oxidative stress genes regarding their responses because of the H_2_O_2_ production [13].

**Fig. 4:**
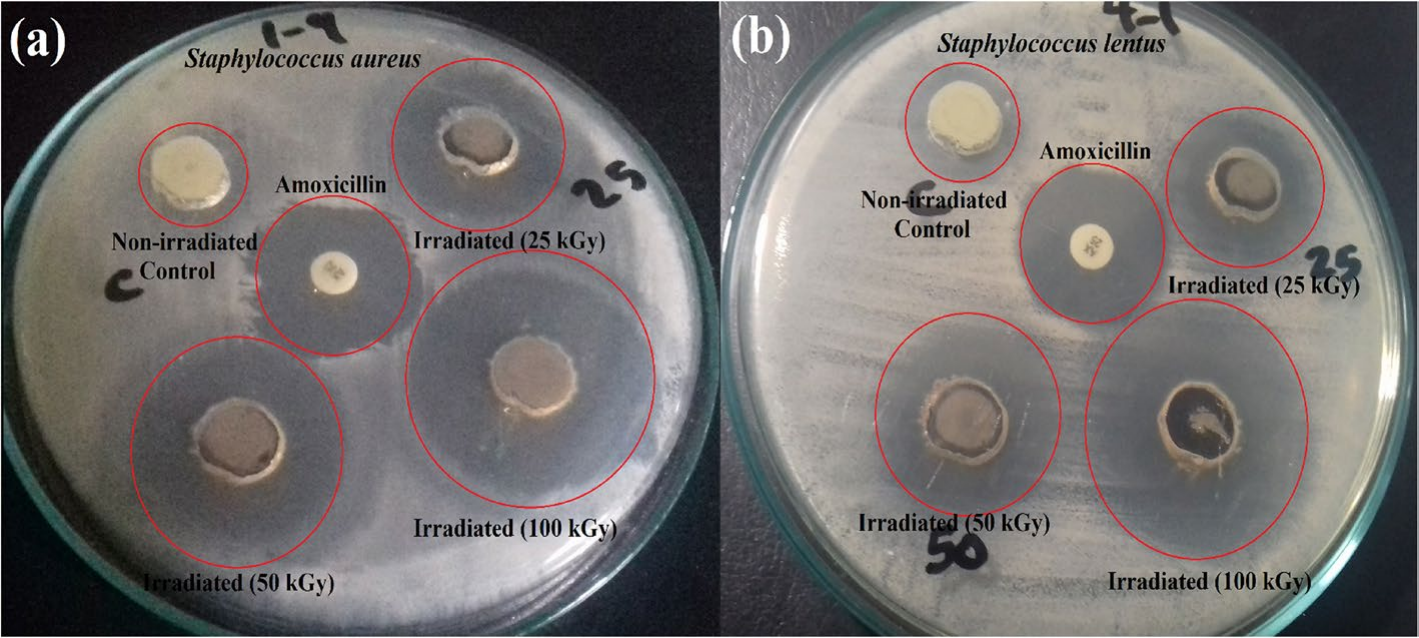
Antimicrobial activities as ZOI (mm), of CdS-Co_x_Ni_1-x_Fe_2_O_4_/SiO_2_/TiO_2_ NPs, irradiated at different gamma-irradiation doses (25, 50 and 100 kGy) against **a** *Staphylococcus aureus*, and **b** *Staphylococcus lentus*

Eventually, after gamma irradiation with doses (25.0, 50.0 and 100.0 kGy), the antimicrobial activity of the synthesized CdS-Co_0.5_Ni_0.5_Fe_2_O_4_/SiO_2_/TiO_2_ NPs nanocomposite was evaluated as noted in Table 2. Gamma-Irradiated CdS-nanocomposite (100 kGy) was more active against *S. aureus* (44.0 mm ZOI; Fig. 4a), and *S. lentus* (41.0 mm ZOI; Fig. 4b), as presented in Table 2.

**Table 2:** Antibacterial activity of gamma-irradiated CdS-Co_x_Ni_1-x_Fe_2_O_4_/SiO_2_/TiO_2_ NPs, against some pathogenic bacteria as ZOI (mm) and MIC (μg/ml)

**Pathogenic bacteria**	**ZOI of non-irradiated CdS-Co**_**x**_**Ni**_**1-x**_**Fe**_**2**_**O**_**4**_**/SiO**_**2**_**/TiO**_**2**_** NPs (mm)**	**ZOI of irradiated CdS-Co**_**x**_**Ni**_**1-x**_**Fe**_**2**_**O**_**4**_**/SiO**_**2**_**/TiO**_**2**_** NPs at 25 kGy (20 ppm) (mm)**	**ZOI of irradiated CdS-Co**_**x**_**Ni**_**1-x**_**Fe**_**2**_**O**_**4**_**/SiO**_**2**_**/TiO**_**2**_** NPs at 50 kGy (20 ppm) (mm)**	**ZOI of irradiated CdS-Co**_**x**_**Ni**_**1-x**_**Fe**_**2**_**O**_**4**_**/SiO**_**2**_**/TiO**_**2**_** NPs at 100 kGy (20 ppm) (mm)**	**MIC of irradiated CdS-Co**_**x**_**Ni**_**1-x**_**Fe**_**2**_**O**_**4**_**/SiO**_**2**_**/TiO**_**2**_** NPs at 100 kGy (20 ppm) (µg/ml)**	▪ ^g^**AX**
*Staphylococcus sciuri*	9.0^a^ ± 0.5507	12.0^a^ ± 0.1527	12.0^a^ ± 0.5000	13.0^a^ ± 0.1527	1.560	20.0
*Staphylococcus vilulinus*	11.0^cd^ ± 0.2886	13.0^bc^ ± 0.2886	19.0^d^ ± 0.1527	25.0^d^ ± 0.5000	0.195	10.0
*Enterococcus columbae*	10.0^bc^ ± 0.2886	12.0^a^ ± 0.5000	14.0^b^ ± 0.2886	19.0^bc^ ± 0.2886	0.780	12.0
*Aerococcus viridians*	10.0^b^ ± 0.1527	12.0^ab^ ± 0.5196	16.0^c^ ± 0.5507	18.0^b^ ± 0.1527	0.780	13.0
*Staphylococcus lentus*	18.0^e^ ± 0.2886	22.0^d^ ± 0.1527	29.0^e^ ± 0.1527	41.0^e^ ± 0.5527	0.0487	24.0
*Staphylococcus aureus*	10.0^bc^ ± 0.5000	29.0^e^ ± 0.5507	35.0^f^ ± 0.5000	44.0^f^ ± 0.5000	0.0243	25.0

Values are means ± SD (*n* = 3)

Data within the groups are analyzed using a one-way analysis of variance (ANOVA) followed by^a, b, c, d, e, f^ Duncan’s multiple range test (DMRT)

^g^*AX* Amoxicillin (antibacterial standard)

Interestingly, the MIC values were decreased by increasing the dose of gamma rays and a superior MIC result was recorded at 0.024 μg/ml against *S. aureus* for CdS-Co_x_Ni_1-x_Fe_2_O_4_/SiO_2_/TiO_2_ NPs nanocomposite irradiated by 100.0 kGy. The enhanced activity of the prepared nanocomposite against all tested microorganisms after gamma irradiation was due to the reduction in their crystallite size after 100 kGy irradiation, as discussed in the published articles [65, 66].

### Antibiofilm activity of gamma-irradiated CdS nanocomposite

The production of biofilm was known in pathogenic microbes that characterized by exo-polysaccharide secretion [67].

The biofilm eradication activity of CdS nanocomposite was assessed against six bacterial species which were isolated from the walls of surgery room (*Staphylococcus lentus*, *Aerococcus* *viridians*, *Staphylococcus sciuri*, *Staphylococcus vilulinus*, *Staphylococcus aureus* and *Enterococcus columbae*). Results revealed the efficiency of CdS nanocomposite to inhibit the biofilm production of most of tested strains. Table 3 showed the antibiofilm activity of gamma irradiated-CdS nanocomposite against the isolated strains.

**Table 3:** Semi-quantitative inhibition % of the biofilm formation for non-treated and treated bacterial pathogens with gamma irradiated-CdS nanocomposite

Bacterial isolate	O.D. of control	O.D. after treatment	Inhibition %
*Staphylococcus lentus*	0.781^f^ ± 0.058	0.170^de^ ± 0.017	78.2
*Aerococcus viridians*	0.100^a^ ± 0.015	0.070^a^ ± 0.017	30.0
*Staphylococcus sciuri*	0.680^e^ ± 0.058	0.097^b^ ± 0.015	85.7
*Staphylococcus vilulinus*	0.197^b^ ± 0.017	0.175^e^ ± 0.058	11.2
*Staphylococcus aureus*	0.320^c^ ± 0.015	0.210^f^ ± 0.015	34.37
*Enterococcus columbae*	0.550^d^ ± 0.017	0.160^c^ ± 0.058	70.9

Values are means ± SD (*n* = 3)

Data within the groups are analyzed using one-way analysis of variance (ANOVA) followed by^a, b, c, d, e, f^ Duncan’s multiple range test (DMRT)

It is worth to mention that, to inhibit the biofilm growth at its constant adhesion stage which is the first step of the antimicrobial behavior, CdS nanocomposite was investigated. The inhibitory percentage depends on several factors including antimicrobial agents’ potential, surface attraction due to the large surface area of the tested nanocomposite, physical characteristics such as particle size, invasion abilities and other chemical factors affecting the interaction of the nanocomposite with the biofilm-producing microbes like surface charge [29].

Fortunately, the tested gamma irradiated-CdS nanocomposite (0.0243 µg/ml) inhibited *S. aureus* growth by more than 98% as shown in MIC results: Table 3. This result might be due to exopolysaccharide synthesis arresting which is the main precursor for biofilm formation. Thus, *S. aureus* biofilm formation had been stopped.

**Fig. 5:**
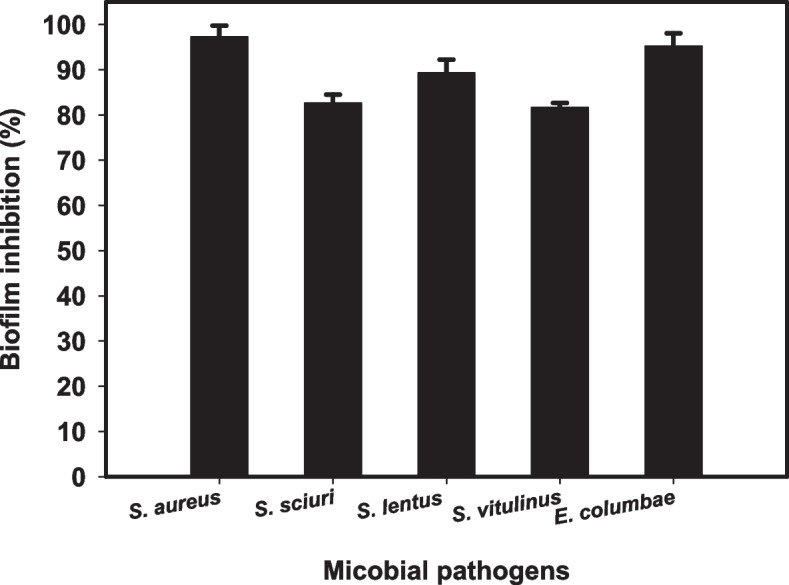
Antibiofilm potential of gamma irradiated-CdS nanocomposite against some pathogenic bacteria inhibition %

Gamma-irradiated-CdS nanocomposite showed highest antibiofilm activity against *Staphylococcus sciuri* (85.7%), *Staphylococcus lentus* (78.2%), and *Enterococcus columbae* (70.9%) as shown in Fig. 5. It was observed that, applying NPs as biofilm inhibitory agents passes by two stages. At the initial stage, microbial cells attachment to the solid substrate could be weakened. Thus, EPS secretion and the progression to the mature stage could be inhibited.

At the second stage, the synthesized nanocomposites can further act on the matured stage of formed biofilm, through penetration leading to destroying the microbial cells and the subsequent formed biofilm dispersion. Consequently, as per our envision, antibiofilm coatings based on some synthesized nanocomposites are promising probes in biofilm imaging, treatment, and eradication.

Furthermore, their minimal toxicity and wide spectrum activity are additional pros. Hence, using semiconductor nanocomposites could not only allow detecting the inhibition process but also their visible monitoring [68].

### Antibacterial potential of gamma-irradiated CdS nanocomposite in liquid media (UV effect)

**Fig. 6:**
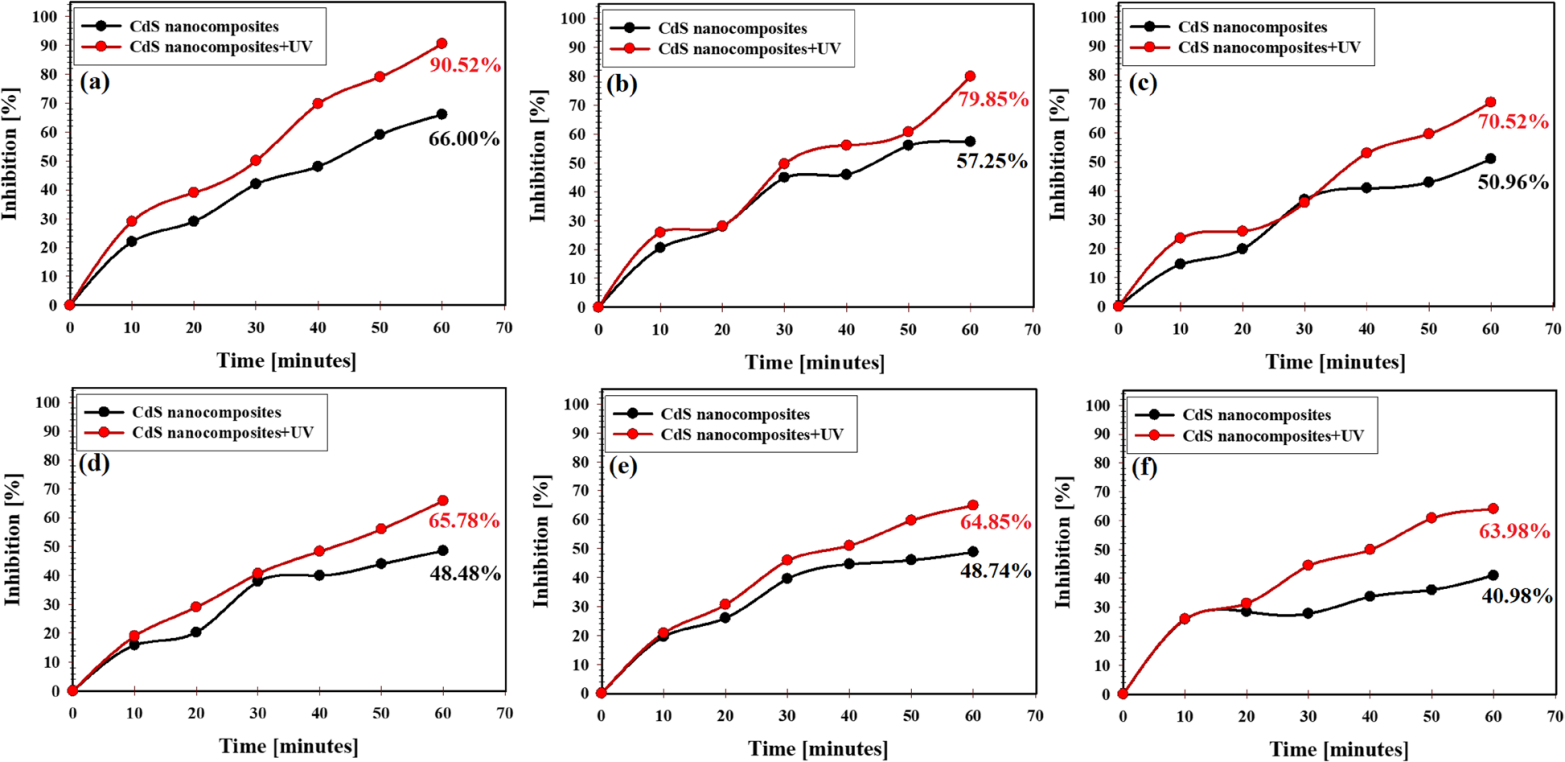
Antimicrobial effect of gamma irradiated-CdS nanocomposite in liquid media under UV-irradiation effect against different pathogenic microbes, where **a** *S. aureus*, **b** *S. lentus,* **c** *S. vilulinus*, **d** *E. columbae*, **e** *A. viridians*, and **f** *S. sciuri*

The comparison results between the hindrance percentage of all tested microbes between gamma-irradiated CdS nanocomposite and UV were presented in Fig. 6. As presented in Fig. 6, the repression percentage of the examined microbial pathogens affected by gamma-irradiated CdS nanocomposite increased through the experiment period meaning that UV-illuminated gamma-irradiated CdS nanocomposite showed antimicrobial activities against all tested microbes more than non-UV-illuminated gamma-irradiated CdS nanocomposite which explains the role of UV-illumination as a synergistic factor in CdS nanocomposite. The synthesized CdS nanocomposite with UV-illumination exhibited maximum inhibition percentage on *S. aureus* (Fig. 6a)*, S. lentus* (Fig. 6b)*, S. vilulinus* (Fig. 6c), *E. columbae* (Fig. 6d), *A. viridians* (Fig. 6e), and *S. sciuri* (Fig. 6f) at the end of the experiment as 90.52%, 79.85, 70.52%, 65.78%, 64.85%, and 63.98%, respectively.

Under UV-irradiation, CdS nanocomposite was further activated, and the formed oxygen species could effectively destroy tested microbial cells. In our study, the prepared CdS nanocomposite possessed an excellent photocatalytic ability and adsorption capability due to their effective UV absorption and surface area, which can enable improved antimicrobial features.

As reported, UV activation of the prepared CdS nanocomposite may produce more OH free radicals. The electron mediator diffuses inside microbial cells and the tested CdS nanocomposite generates free OH radicals which in return destroys microbial cells through microbial coenzymes destruction and reduction of their contents [69]. As reported, many metal oxide NPs possess net positive charge on their surface while negative charge was found in the outer layer of the tested microbial cells which simply enables the electrostatic attraction leading to the oxidization of cell components and subsequent microbial cell lysis [70]. On the other hand, nanomaterials-based composites could attack genetic materials (DNA) and the main cellular catalysts via electron-providing groups (indoles, hydroxyls, amides, thiols, and carbohydrates) [55].

### Growth curve method

**Fig. 7:**
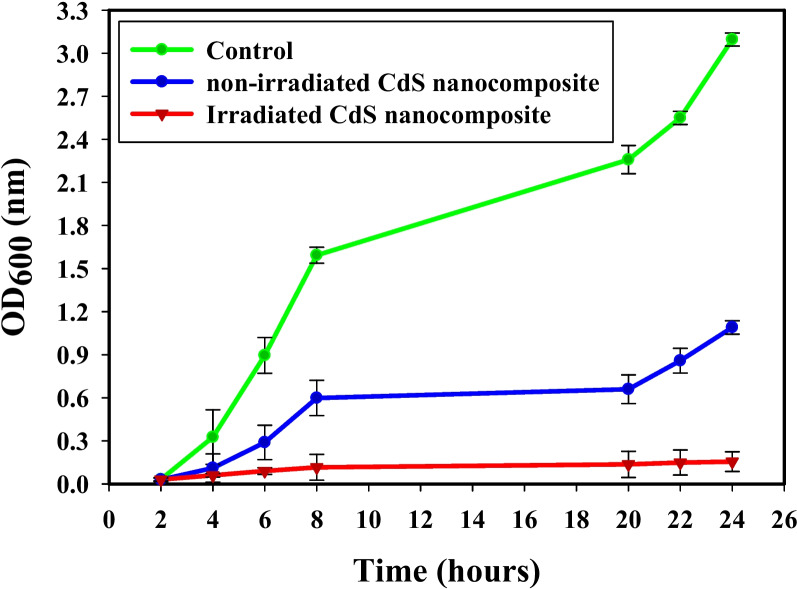
The effect of non-irradiated, irradiated-CdS nanocomposite on the growth curve of *S. aureus*

The influence of non-irradiated and irradiated CdS nanocomposite on *S. aureus* growth had presented in Fig. 7. *S. aureus* growth in the control sample happened quickly, with the most potent optical density at λ = 600 nm (OD_600_) value having arrived at about 3.09 nm. After adding non-irradiated CdS nanocomposite, changes were detected, and OD_600_ was calculated to be 1.09 nm. Indifference, the OD_600_ value of irradiated CdS nanocomposite showed the weakest results (0.115 nm), and showing the repression impact on the growth of *S. aureus*. Irradiated CdS nanocomposite display further suppressing power rather than non-irradiated CdS nanocomposite that the unique synergistic effects may define, and the effect of small particle sized as described in our previous study [1].

Previous findings have often described the creation of some ROS on the surface of NPs [71, 72]. Irradiated CdS nanocomposite produces ROS, which cause protein oxidation, DNA damage, and lipid peroxidation, which can destroy bacteria [73]. Additionally, the metal ions generated by irradiated CdS nanocomposite (Cd^2+^) have a positive charge in contrast to *S. aureus* membrane, which maintains a negative charge. This means that they develop in direct relation to stop the replication of bacterial DNA, denaturation of proteins, and bacterial cell breakdown [74]. The greater stiffness of the membrane of bacterial cells may be the cause of the Gram-positive bacteria's enhanced responsiveness to the synthesized NPs [75].

The size, form, and charge on the surface of irradiated CdS nanocomposite are other potential causes that may make them more advantageous to link with Gram-positive bacteria. According to Xu et al*.* [76]*,* some synthesized NPs, following 80 min of UV irradiation, ruptured the *E. coli* membrane, indicating that disinfection was complete. Numerous studies showed that the majority of NPs showed antibacterial potential against different bacterial strains, including *E. coli*, and *S. aureus* [1, 20, 45, 77–82].

### Bacterial protein leakage investigation

**Fig. 8:**
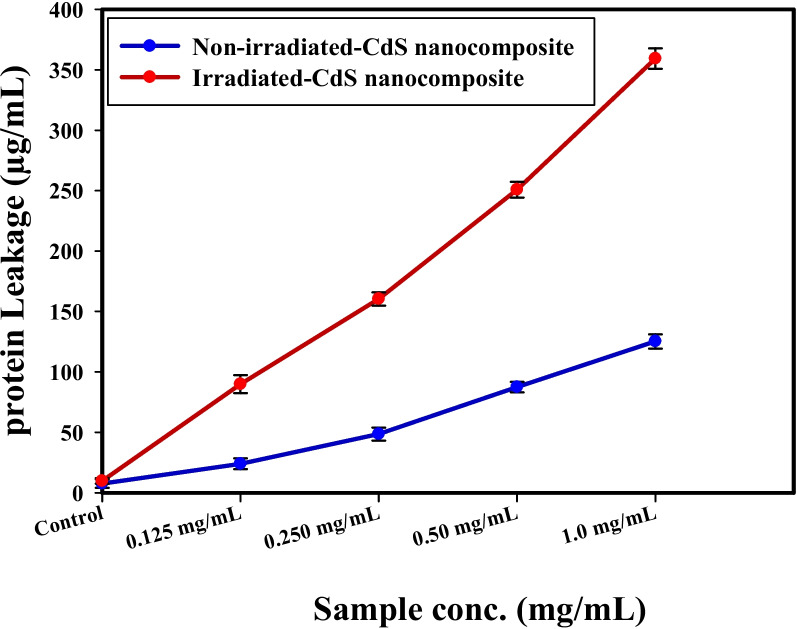
The effect of non-irradiated, and irradiated-CdS nanocomposite on the protein leakage from *S. aureus* cell membranes

In the treated *S. aureus* suspension, the amounts of proteins discharged were determined by the Bradford method [83]. From Fig. 8, positively, the amount of bacterial protein removed is directly proportional after increasing the concentration of non-irradiated, and irradiated CdS nanocomposite (at different concentrations) and counted to be 125.28, and 359.25 µg/ml following the treatment with non-irradiated, and irradiated CdS nanocomposite, respectively (1.0 mg/mL), which demonstrates the antibacterial features of the irradiated CdS nanocomposite and describes the appearance of holes in the *S. aureus* membrane, which help in making the proteins bleed out from the *S. aureus* cytoplasm.

The presented results indicated that bacterial protein removed is directly proportional after increasing the concentration of enhanced the dissolving of *S. aureus* membrane permeability. The main cause for the repression of the bacterial mass is the positive effect upon the membrane permeability regarding the protein leakage. Corresponding references such as [84, 85] define similar results when combined NPs, which showed concentration-dependence for the dislodgement in the bacterial membrane and suggested leakage of bacterial intracellular organelles into the extracellular cell structure.

According to Paul, et al. [86] the difference in the bacterial membrane permeability was established in the rate difference in the linked electric conductivity. The protein leakage test is a crucial technique used to evaluate the structural integrity of any microorganisms. Over time, the leakage evolved into typical microbial destruction, and the release of cell components led to cell death.

### Reaction mechanism determination by SEM imaging

**Fig. 9:**
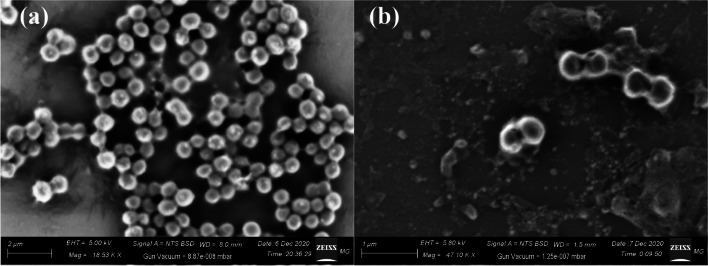
SEM of *S. aureus* where **a** Normal bacterial cells without gamma irradiated-CdS nanocomposite treatment, and **b** Depressed and deformed bacterial cell after gamma irradiated-CdS nanocomposite treatment

SEM imaging analysis had been conducted to explain the potential antimicrobial mechanism against *S. aureus*, see Fig. 9. The SEM examination regarding the control bacteria in the lack of gamma irradiated-CdS nanocomposite conferred bacterial colonies regularly extended with standard normal surface and semi-formed biofilm, Fig. 9a. Upon gamma irradiated-CdS nanocomposite treatment, remarkable morphological abnormalities were recognized in *S. aureus* (Fig. 9a), including the total lysis of the exterior surface supported by deformations of the *S. aureus* cells. Additionally, the gamma irradiated-CdS nanocomposite created the complete lysis of the bacterial cell and cell distortion, reducing the total viable number. Finally, the biofilm extension was limited (Fig. 9b).

**Fig. 10:**
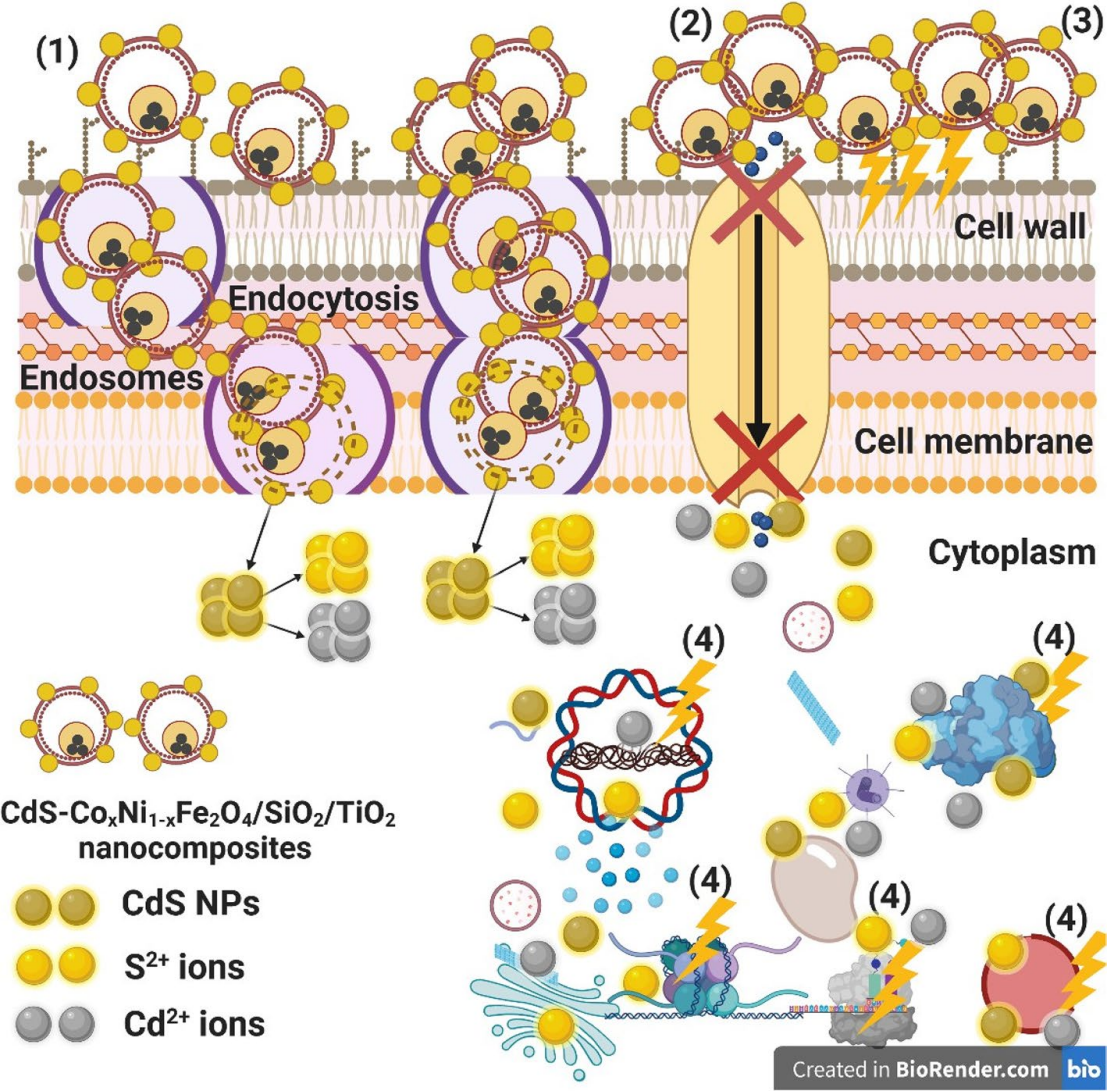
Schematic representation regarding the four prominent ways of antimicrobial potential of CdS-Co_x_Ni_1-x_Fe_2_O_4_/SiO_2_/TiO_2_ nanocomposites, where (1) CdS-Co_x_Ni_1-x_Fe_2_O_4_/SiO_2_/TiO_2_ nanocomposites adhere to and wrapped the microbial cell surface and results in CdS NPs release which causing membrane damage and altered transport activity, (2) CdS-Co_x_Ni_1-x_Fe_2_O_4_/SiO_2_/TiO_2_ nanocomposites block the ions transport from and to the microbial cell, (3) CdS-Co_x_Ni_1-x_Fe_2_O_4_/SiO_2_/TiO_2_ nanocomposites increase the ROS (due to the activation after gamma irradiation) leading to cell damage, and (4) CdS-Co_x_Ni_1-x_Fe_2_O_4_/SiO_2_/TiO_2_ nanocomposites penetrate inside the microbial cells and interact with cellular organelles and biomolecules, and thereby affect respective cellular machinery. CdS NPs may serve as a vehicle to effectively-deliver Cd^2^^+^, and S^2^^+^ ions to the microbial cytoplasm and membrane, where proton motive force would decrease the pH to be less than 3.0 and therefore improve the release of Cd^2^^+^, and S^2^^+^ ions. "Created with BioRender.com''

The schematic illustration in Fig. 10 shows the potential antimicrobial mechanism. There were terrific superior mechanisms like Reactive Oxygen Species (ROS) distribution such as superoxide anion; O_2_^−^, as mentioned in the recent references [26, 87], the succession of CdS (surface loaded), inside the pathogenic microbes, and an alkaline tendency was admitted to show the antimicrobial action mechanism. Our suggested action mechanism started with the adhesion of the nanocomposite to the outer surface of *S. aureus*. Then, Cd^2+^, and S^2+^ ions penetrate the tested bacterial cells and destroyed their biological molecules. After that, cellular toxicity due to oxidative tension and the generated ROS increased as suggested in the recent publications [26, 88, 89], which could change the microbial morphology, reduce the microbial membrane permeability and provide the residence of oxidative stress genes concerning their responses because of the H_2_O_2_ generation as confirmed in the recent papers [26, 29, 87, 89].

Finally, the ionic species were formed (Cd^2^^+^, and S^2^^+^ ions) in the acidic medium, leading to cellular toxicity and genotoxicity because of the interaction among the negatively charged vital organs [78, 81]. The major observed impacts are hole penetration of microbial cell walls which subsequently initiate cell death.

### MB degradation by the prepared samples

**Fig. 11:**
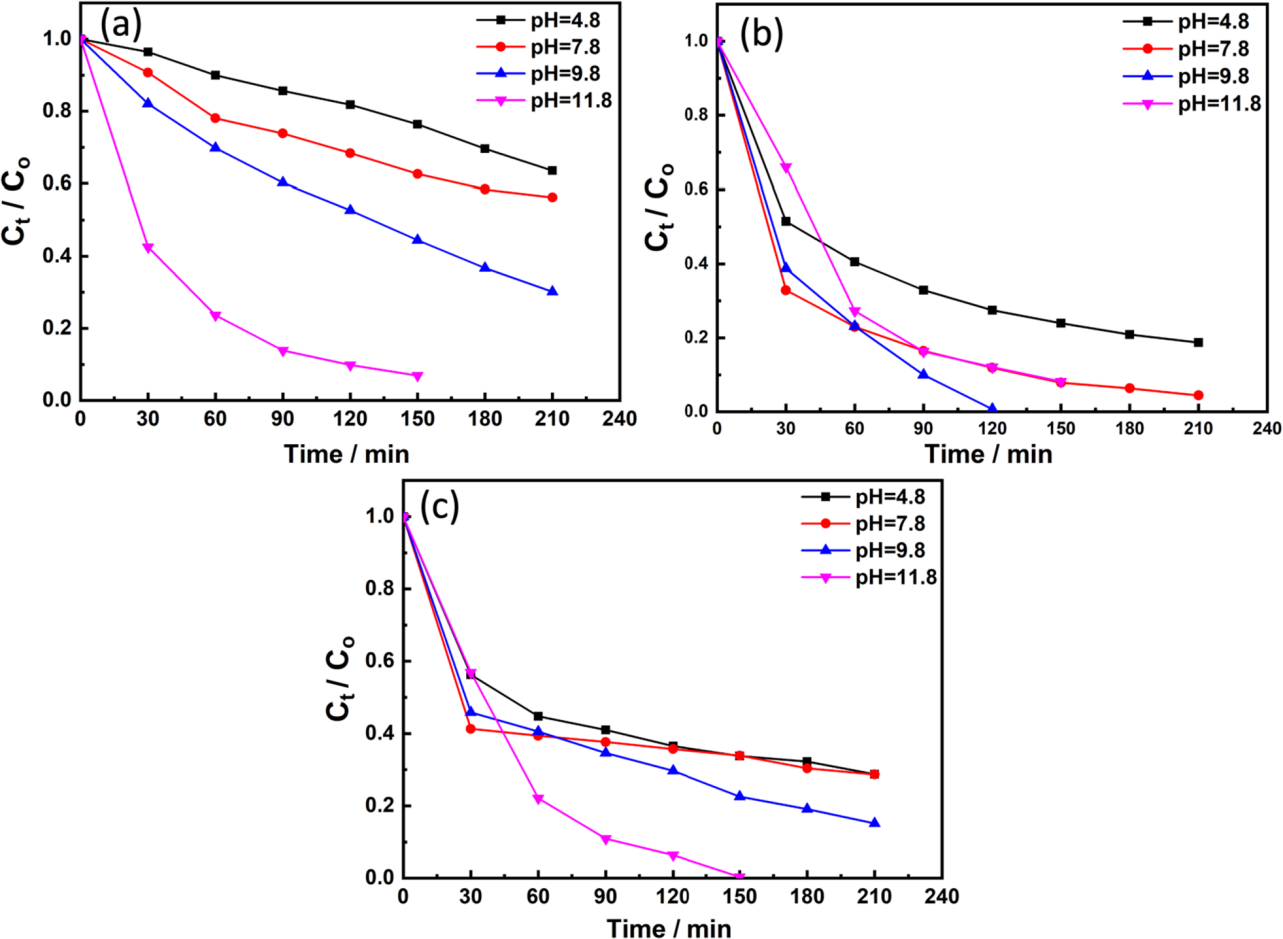
MB photocatalytic degradation by **a** composite matrix, **b** CdS NPs, and **c** CdS-loaded nanocomposite

Methylene blue dye was used as a model for evaluating the photocatalytic activity of the prepared materials (nanocomposite, CdS NPs and CdS-loaded nanocomposite) under light irradiation (Fig. 11).

It is clear that over a wide range of pH values (4.8. 7.8, 9.8, and 11.8), the prepared nanocomposite can’t do complete degradation of the dye after 210 min under illumination (Fig. 11a). However, at pH ~ 11.8 its photocatalytic activity was enhanced and became faster as will be discussed later. In case of CdS NPs, it is obviously seen that the catalytic activity at the same pH ranges is higher compared to the prepared nanocomposite (Fig. 11b).

This is due to the smaller band gap of the prepared CdS NPs (about 2.4 eV). This gave the advantages of absorbing the visible light which constitutes the largest part of the used light for illumination. Once the CdS NPs was added to the surface of the prepared nanocomposite, it acts as photosensitizer to composite nanoparticles. This explain why the photocatalytic activity of the CdS loaded nanocomposite was enhanced and MB dye was completely degraded after 150 min at pH ~ 11.8 (Fig. 11c), however in the case of CdS NPs and the pure nanocomposite the degradation percent was about 90% at the same pH.

It is well known that the concentrations of H^+^ and OH^-^ ions change when the pH changes. Also, it is generally known that photocatalytic reaction, which is caused by the interaction of semiconductor nanocatalysts and H_2_O with light radiation, is a sort of oxidation reaction that uses the OH radical as a promoter. As a result, one of the key influencing factors in this kind of reaction is the OH radical abundance and concentration. Increased pH causes an increase in the concentration of OH ions, which are easily converted to hydroxyl radicals on the surface of semiconductor nanocatalysts (nanocomposite, CdS NPs and CdS-loaded nanocomposite) in the presence of created photo holes [90]. As a result, OH ions act as a photo-hole absorber, and the rate of the photocatalytic process increases as the concentration of OH increases [91].

All samples showed outstanding activity at the alkaline pH values (9.8 for CdS NPs) and (11.8 for the bare composite matrix and the CdS-loaded nanocomposite). At this pH values, all samples possessed higher Z-potential value indicating their colloidal stabilities with net negative surface charges (- 44.3 mV and -51 mV) for CdS and the bare composite matrix, respectively. As MB dye possesses net positive charge (cationic dye), this enhanced the electrostatic interaction between them.

It is worth mentioned that, compared to bare composite matrix, CdS-loaded sample showed higher photocatalytic activity, which could be attributed to the synergetic effect of CdS NPs and the composite matrix, which allowed better charge separation and elongation of the lifetime of the photo generated electron hole pairs.

### The proposed mechanism

**Fig. 12:**
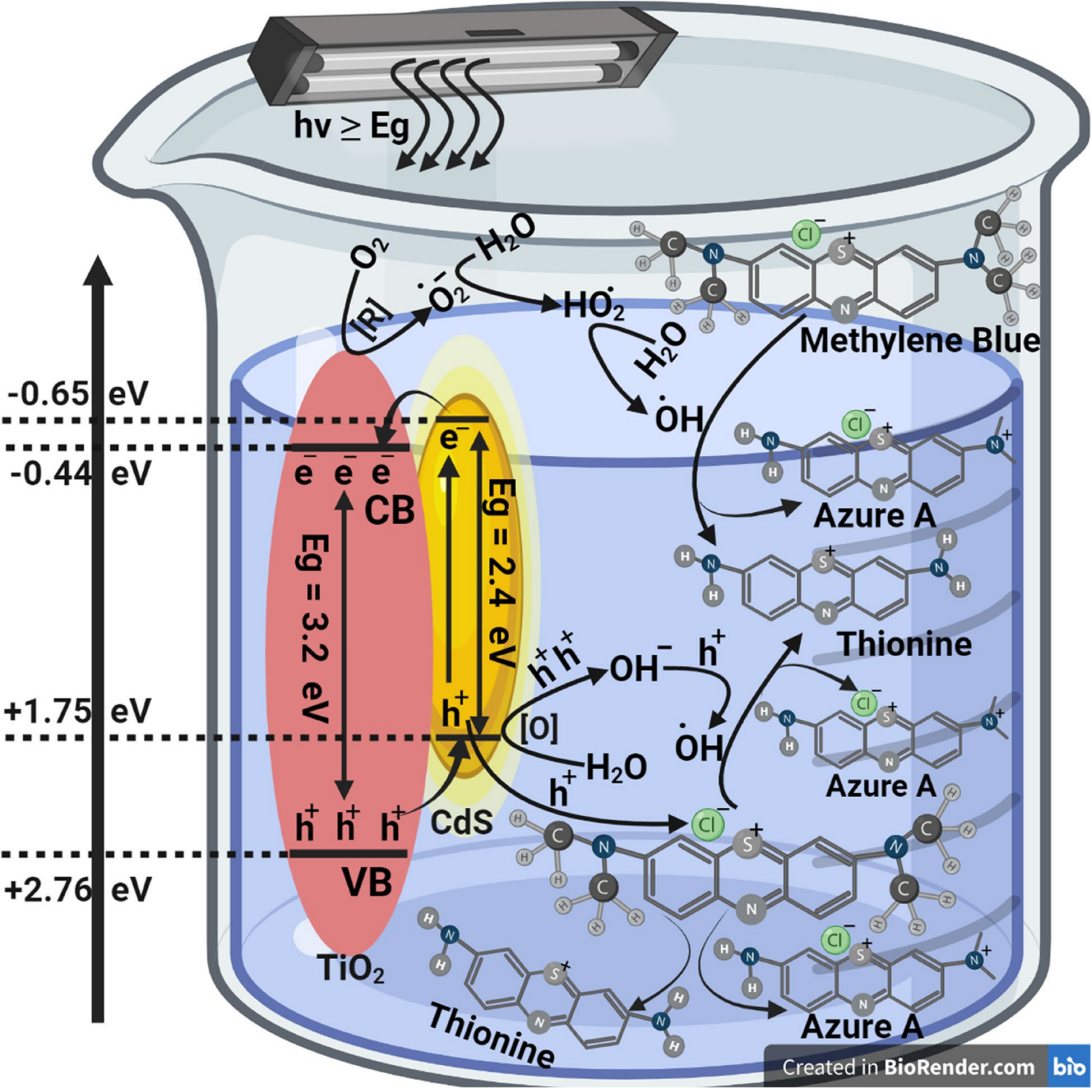
MB photocatalytic degradation reaction mechanism "created with BioRender.com''

The suggested dye degradation mechanism over CdS loaded nanocomposite under light illumination was presented in Fig. 12. As CdS NPs are visible light active material, once it was illuminated with light, it absorbs the light and some of the valence band (VB) electrons are excited and jumped to the conduction band (CB) leaving behind holes at the VB. As the CB of CdS NPs (- 0.56 eV) are less negative than the CB of TiO_2_ NPs (- 0.44 eV), (outer layer of the composite matrix), excited electrons are transferred to the CB of TiO_2_ layer. Thus, the conduction band of TiO_2_ will act as electron center and from one side the electrons can interact with oxygen molecules and highly reactive species will be produced such as $\mathrm O_2^{\bullet-}$. This highly reactive species can facilitate the reduction of MB dye molecules to beak it. At the other side, VB of TiO_2_ (+ 2.76 eV) are more positive than the VB of CdS NPs (+ 1.75 eV), the holes produced at the nanocomposite will be transferred to the opposite direction to the CdS NPs and will react with the water molecules at the surface forming a highly active species of $\mathrm{OH}^\bullet$.This active free radical can oxidize the MB dye molecules forming degradation products which can be (Azure a, b, c and thionine) [92]. Due to the transfer of electron from the VB of CdS NPs to the CB of the composite, this led to improved charge separation, and this is explaining why the photocatalytic activity was enhanced after loading CdS NPs on the nanocomposite matrix.

The reactions of the electron–hole in Cds loaded nanocomposite can be described as in the following Eqs. (1, 2, 3, 4 and 5):

1 $${\mathbf{C}\mathbf{o}}_{\mathbf{0}\boldsymbol{.}\mathbf{5}}\mathbf{N}\mathbf{i}_{\mathbf{0}\boldsymbol{.}\mathbf{5}}{\mathbf{F}\mathbf{e}}_{\mathbf{2}}{\mathbf{O}}_{\mathbf{4}}/{\mathbf{S}\mathbf{i}\mathbf{O}}_{\mathbf{2}}/{\mathbf{T}\mathbf i\mathbf{O}}_{\mathbf{2}}/\mathbf{C}\mathbf{d}\mathbf{S}+\mathbf{h}\gamma\rightarrow\mathbf e^-+\mathbf h^+$$

2 $$\mathbf e^-+\mathbf o\mathbf x\mathbf y\mathbf g\mathbf e\mathbf n\boldsymbol\;\mathbf m\mathbf o\mathbf l\mathbf e\mathbf c\mathbf u\mathbf l\mathbf e\mathbf s\rightarrow\mathbf O_{\mathbf2}^{\bullet-}$$

3 $${\mathbf{h}}^{+}\boldsymbol{ }+\boldsymbol{ }{\mathbf{H}}_\mathbf{2}\mathbf{O}\to {\mathbf{h}}^{+}+{\mathbf{O}\mathbf{H}}^{-}$$

4 $$\mathbf h^++{\mathbf O\mathbf H}^-\rightarrow{\mathbf O\mathbf H}^\bullet$$

5 $${\mathbf O\mathbf H}^\bullet+\mathbf O_{\mathbf2}^{\bullet-}+\mathbf M\mathbf B\rightarrow\mathbf D\mathbf e\mathbf g\mathbf r\mathbf a\mathbf d\mathbf a\mathbf t\mathbf i\mathbf o\mathbf n\boldsymbol\;\mathbf p\mathbf r\mathbf o\mathbf d\mathbf u\mathbf c\mathbf t\mathbf s\left(\mathbf A\mathbf z\mathbf u\mathbf r\mathbf e\mathbf a,\mathbf b,\mathbf c\boldsymbol\;\mathbf a\mathbf n\mathbf d\boldsymbol\;\mathbf t\mathbf h\mathbf i\mathbf o\mathbf n\mathbf i\mathbf n\mathbf e\right)$$

### Reaction kinetics

The term "photocatalysis of dyes" refers to the heterogeneous catalytic interaction between the dye and semiconductor nanocatalysts (nanocomposite, CdS NPs and CdS-loaded nanocomposite), and the oxidation reaction is aided by the OH^˙^ radicals produced by the reaction of the semiconductor and H_2_O molecule under light illuminations. The catalyst concentration, pH, and temperature of the dye solution all have a significant impact on the degradation process. The heterogeneous photocatalytic reaction must adhere to some sort of reaction kinetics, just like any other chemical reaction [93]. When a reaction first begins and continues for up to few minutes, it often follows pseudo-first order reaction kinetics.

**Fig. 13:**
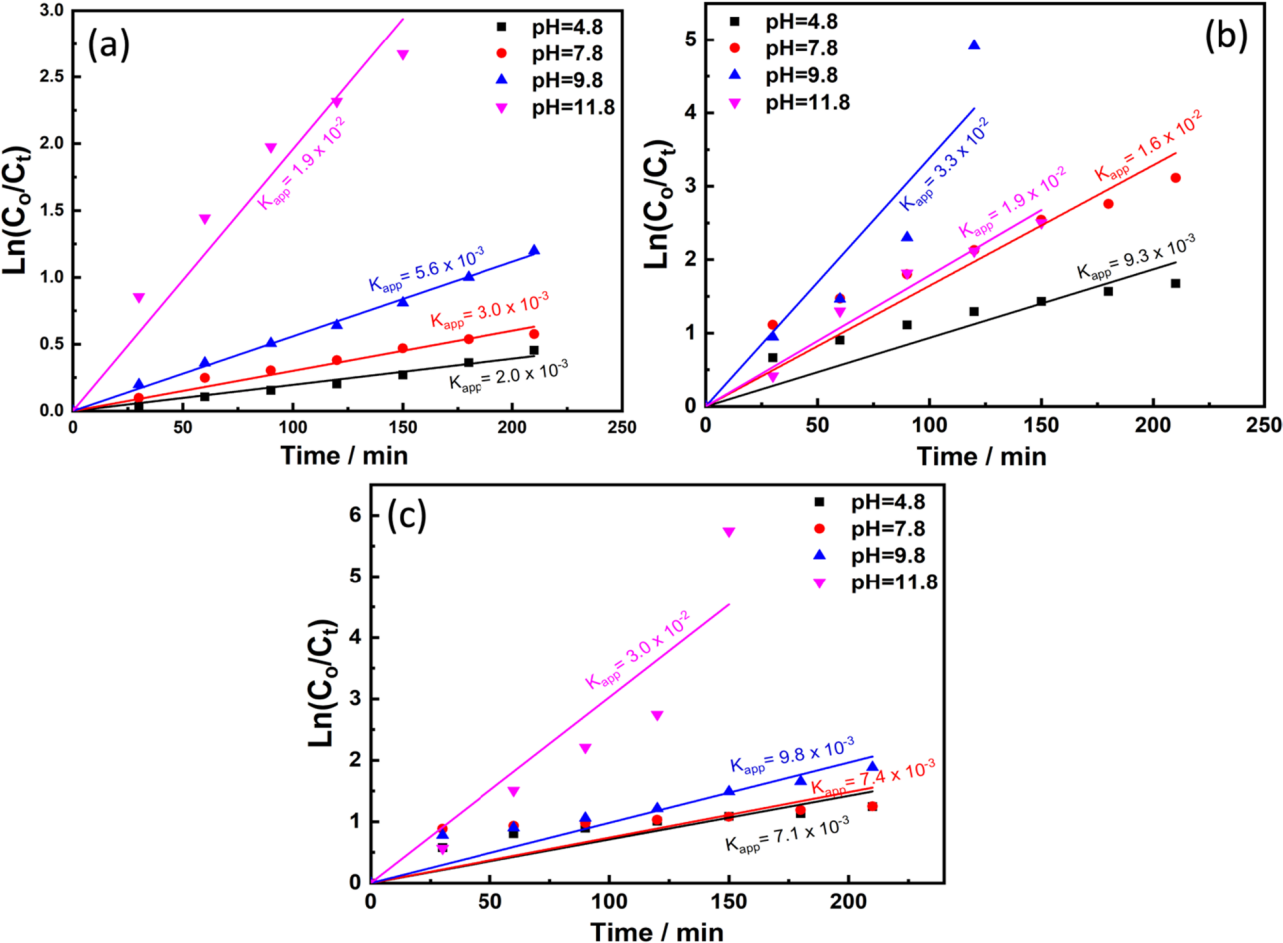
Reaction kinetics and apparent rate constant for MB photocatalytic removal by **a**) composite matrix NPs, **b** CdS NPs, and **c** CdS-loaded nanocomposite

MB photocatalytic degradation kinetics with initial dye concentration of 1 × 10^–5^ M and catalyst weight of 100 mg/L under light illumination was studied and evaluated through plotting ln(C_t_/C_0_) on Y axis versus time on the X axis as seen in Fig. 13(a-c).

The linear structure of the obtained plots in case of all catalysts at all pH ranges reveals that the photocatalytic reactions followed the pseudo first order model shown in Eq. (6).

6 $${\varvec{L}}{\varvec{n}}\boldsymbol{ }\left(\frac{{\varvec{C}}{\varvec{t}}}{{\varvec{C}}{\varvec{o}}}\right)=-{{\varvec{K}}}_{{\varvec{a}}{\varvec{p}}{\varvec{p}}}{\varvec{t}}$$

*Where C*_*t*_ *is the dye concentration at specific time (t) of illumination C*_*0*_ *is the dye concentration before illumination and K*_*app*_ *is the rate constant (min* ^*−1*^ *) of the photocatalytic degradation reaction.*

It was found that the rate constants at pH 4.8, 7.8, 9.8, and 11.8 in case of using the bare nanocomposite as photocatalyst are 2 × 10^–3^, 3 × 10^–3^, 5.6 × 10^–3^ and 1.8 × 10^–2^ respectively. While in case of CdS NPs, the rate constants are 9.3 × 10^–3^, 1.6 × 10^–2^, 1.9 × 10^–2^ and 3.3 × 10^–2^ at the pH ranges respectively.

However, in case of Cds loaded nanocomposite particles, the rate constants are 7.1 × 10^–3^, 7.4 × 10^–3^, 9.8 × 10^–3^ and 3 × 10^–2^ at the pH ranges, respectively. These values confirmed that the photocatalytic degradation reaction became faster when comparing the bare nanocomposite with the CdS loaded nanocomposite.

## Conclusion

A facile process was used to prepare CdS-loaded nanocomposite (Co_0.5_Ni_0.5_Fe_2_O_4_/SiO_2_/TiO_2_/CdS) through a layer-by-layer method followed by a simple impregnation route. The SEM, and EDX analyses results indicated the efficient formation and purity of the investigated samples. Also, HR-TEM images confirmed the conjugation of CdS and Co_0.5_Ni_0.5_Fe_2_O_4_/SiO_2_/TiO_2_ nanocomposite. The photocatalytic experiments confirmed the enhanced, stable, and outstanding effect towards MB degradation**.** Once the CdS NPs were loaded on the surface of the prepared nanocomposite, they acted as photosensitizer to composite nanoparticles. This explain why the photocatalytic activity of the CdS loaded nanocomposite was improved and MB dye was completely degraded after 150 min at pH ~ 11.8. However, in the case of bare CdS NPs and pure nanocomposite, the degradation percent was about 90% at the same pH**.** The developed CdS, and CdS-loaded nanocomposite samples had been tested for their antibacterial potential against selected pathogens (separated from surfaces and exteriors of the medical operating places) through the agar-disc diffusion method. The tested samples showed a positive potency to a wide spectrum of bacteria. In special result, CdS-nanocomposite (20 ppm) have the most powerful influence against all the examined microbes. Eventually, after gamma irradiation with different doses, the antibacterial activity of the synthesized CdS-Co_0.5_Ni_0.5_Fe_2_O_4_/SiO_2_/TiO_2_ nanocomposite (100 kGy) was more active against *S. aureus* (44.0 mm ZOI), and *S. lentus* (41.0 mm ZOI). In antibiofilm assay, gamma irradiated-CdS nanocomposite showed highest antibiofilm activity against *S. sciuri* (85.7%), *S. lentus* (78.2%), and *E. columbae* (70.9%)**. **In growth curve assay, the OD_600_ value gamma irradiated-CdS nanocomposite showed the weakest results (0.115 nm), and showing the repression impact on the growth of *S. aureus*. In UV experiment, CdS-nanocomposite with UV-illumination exhibited maximum inhibition percentage on *S. aureus, S. lentus, S. vilulinus*, *E. columbae*, *A. viridians*, and *S. sciuri* at the end of the experiment as 90.52%, 79.85, 70.52%, 65.78%, 64.85%, and 63.98%, respectively. In the protein leakage method, the quantity of bacterial protein eliminated is immediately corresponding to the raising in the concentration of non-irradiated and irradiated CdS nanocomposite (at various concentrations). After treatment with non-irradiated and irradiated CdS nanocomposite, respectively (1.0 mg/mL), the results were counted to be 125.28 and 359.25 µg/ml, which illustrates the antibacterial properties of the irradiated CdS nanocomposite. This discusses the formation of pores in the *S. aureus* membrane that aid in causing the proteins to leak out of the cytoplasm of the organism. In the absence of CdS-nanocomposite, the SEM analysis of the untreated bacteria revealed that bacterial colonies were normally expanded with a typical surface and a semi-formed biofilm. After being exposed to CdS nanocomposite, *S. aureus* exhibited striking morphological aberrations, including entire lysis of the external surface accompanied by cell deformation. The total amount of viable bacteria was decreased by the CdS-nanocomposite's full lysis and cell deformation. The biofilm's expansion was also minimal.

### Supplementary Information

**Additional file 1: Figure S1.** Histogram of the particle size distribution with Gaussian fitting of CdS-loaded nanocomposite.

## Data Availability

The datasets used and/or analyzed during the current study are available from the corresponding author on reasonable request.
