# Supplementary material for: Potential antibacterial, antibiofilm, and photocatalytic performance of gamma-irradiated novel nanocomposite for enhanced disinfection applications with an investigated reaction mechanism
Source: BMC Microbiol. 2023 Sep 26;23:270. doi: 10.1186/s12866-023-03016-3 (PMC10521429; doi:10.1186/s12866-023-03016-3)
Supplement: Supplementary file 1 — Additional file 1: Figure S1. Histogram of the particle size distribution with Gaussian fitting of CdS-loaded nanocomposite. [file 12866_2023_3016_MOESM1_ESM.docx]

**
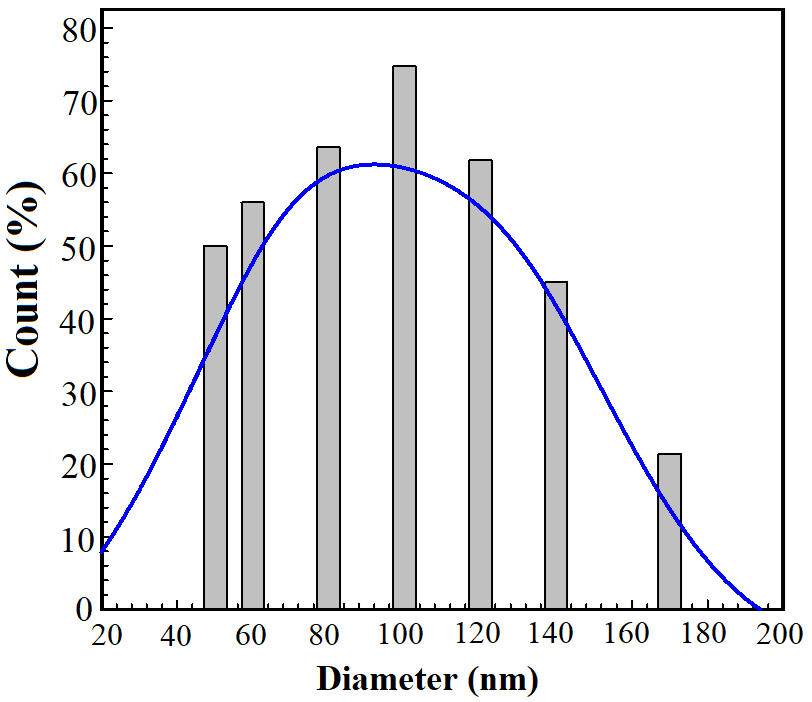
**

**Figure S1: histogram of the particle size distribution with Gaussian fitting of CdS-loaded nanocomposite.**
